# Resveratrol Enhances the Anti-Cancer Effects of Cis-Platinum on Human Cervical Cancer Cell Lines by Activating the SIRT3 Relative Anti-Oxidative Pathway

**DOI:** 10.3389/fphar.2022.916876

**Published:** 2022-07-05

**Authors:** Bin Jiang, Qi Tian, Chuqiang Shu, Jing Zhao, Min Xue, Shujuan Zhu

**Affiliations:** ^1^ Department of Obstetrics and Gynecology, The Third Xiangya Hospital of Central South University, Changsha, China; ^2^ Department of Obstetrics and Gynecology, Hunan Provincial Maternal and Child Health Care Hospital, Changsha, China; ^3^ National Health Commission Key Laboratory of Birth Defects Research, Prevention and Treatment, Hunan Provincial Maternal and Child Health Care Hospital, Changsha, China

**Keywords:** cervical cancer, resveratrol, cis-platinum, SIRT3, anti-oxidative pathway

## Abstract

**Background:** Cervical cancer exerts considerable mortality in the world. The combinations of chemotherapy with cis-platinum were the first-line treatment in late-stage cervical cancer but may cause severe adverse effects. Resveratrol (RES, 3,5,4′-trihydroxy-trans-stilbene) is a phytoalexin, and it showed anti-cancer effects but with low toxicity and side effects. Herein, we examined the anti-cancer effects of cis-platinum combined with RES in human cervical cancer cell lines.

**Methods:** The antiproliferative effect was examined by cell counting and short-term MTT assay. Cell apoptosis was detected. The cell cycle distribution was determined by flow cytometry. Intracellular reactive oxygen species and mitochondrial transmembrane potential change were observed and calculated by confocal microscopy. The Si-RNA interference of *SIRT3* in cancer cells was performed. Protein expression was checked by Western blotting.

**Results:** RES inhibited the growth of SiHa cell lines, and it significantly enhanced the cis-platinum-induced cell apoptosis and cell cycle arresting in 48 h. The activation of the SIRT3 relative anti-oxidative pathway was proved to be the reason for the enhanced anti-cancer effects of cis-platinum and RES combination. Si-RNA interference of *SIRT3* compromised the anti-cancer effect of cis-platinum and RES combination. Furthermore, the silencing of *SIRT3* RNA inhibited the expression of the anti-oxidant enzyme (MnSOD, GPx, SOD-1, and CAT) and decreased the generation of H_2_O_2_ in the cis-platinum and RES combination group.

**Conclusion:** RES enhances the anti-cancer effects of cis-platinum on SiHa cells by activating the SIRT3 relative anti-oxidative pathway. RES may act as a potential synergistic agent and be useful in the treatment of cervical cancer.

## Introduction

Cervical cancer is the most common type of cancer in women globally, and it ranks second in the incidence of cancer in developing countries (Motoki et al., 2015; Schiffman, 2017). The treatment of advanced cervical cancer involves chemotherapy and/or radiotherapy. The adjuvant cis-platinum (cis-DDP)-based chemotherapy was the first-line treatment (Rosen et al., 2017; Aghili et al., 2018). However, the most currently available chemotherapeutic agents are commonly associated with adverse effects and may impact the life quality of the patients negatively (Mallmann and Mallmann, 2016).

Resveratrol (RES, 3,5,4′-trihydroxy-trans-stilbene) is a natural antioxidant polyphenol compound, and it was found in many eatable plant species (Salehi et al., 2018). In recent years, studies have proved the anti-cancer activity of RES in many tumors with few adverse effects (Ko et al., 2017). RES alone showed the ability to suppress the transcription and expression of HPV E6 and E7 genes and inhibited the progression of cervical cancer cell lines (Sun et al., 2021). Although RES was proved to inhibit the growth of cervical cancer cells (Kim et al., 2012), RES’s anti-cancer effects through its anti-oxidative nature were understudied, especially when RES was combined with cis-DDP.

The antioxidant activities of RES were related to the activation of sirtuin (SIRT) proteins (Bagul et al., 2018). SIRT-3 was proved to regulate the activity of mitochondrial antioxidant enzymes and the high oxidation state in tumor cells (Torrens-Mas et al., 2017). It is not fully understood whether the SIRT-3 relative anti-oxidative pathway may play a role in the anti-cancer effect of RES and cis-DDP combination. Herein, the anti-cancer effects of RES and cis-DDP combination were examined against the SiHa cell line, and an attempt was made to study the SIRT-3-related mechanism.

## Methods

### Cell Culture and Small Interfering RNA Transfection

SiHa Cervical cancer cell lines were processed from American Type Culture Collection (Manassas, VA, United States). The cells were cultured in Dulbecco’s modified Eagle’s medium (DMEM) with 10% fetal bovine serum (FBS) (Thermo Fisher Scientific, Inc. Waltham, MA, United States), 2 mM glutamine, and antibiotics (100 μg/ml streptomycin and100 Uml penicillin). The cells were maintained in a CO_2_ incubator at 37°C with 98% humidity and 5% CO2. For the SIRT3 RNA silencing, nonspecific control siRNA or SIRT3 siRNA was transfected using the siLentFect Lipid Reagent (Bio-Rad, Hercules, CA, United States), according to the manufacturer’s instructions. The transfection was confirmed with RT-PCR and Western blotting.

### Cell Viability and MTT Assays

The viability of the SiHa cervical cancer cell lines was examined by cell counting assay with Trypan blue staining. The inverted microscope was used for observing the growth and morphological changes of cells in each group. The expression of proliferating cell nuclear antigens (PCNA) was labeled by the monoclonal anti-PCNA antibody PC10 and detected by the immunohistochemistry method (Lu et al., 2019). MTT assays were performed to assess the growth inhibition of treatments and calculate the optimum concentration for the agents. In brief, 6×10^5^ cells were seeded in 96-well plates and incubated for 48 h at 37°C and 5% CO_2_. Various concentrations of RES and/or cis-DDP were added to each well at 24 h. Before the end of each incubation period, 10 μL of the MTT labeling reagent was added (final concentration 0.5 mg/ml) to each well and allowed for incubation for 4 h. The absorbance was measured at 570 nm calibration by using a microplate (ELISA) reader, and an inhibition curve was made to calculate the cell inhibition rate. Calculation of the drug interaction index (CDI) was made, and a synergistic effect was defined when CDI<1.

### AO/PI Staining for Apoptosis and Cell Cycle Analysis

For AO/PI staining (Mishra et al., 2019), the cervical cancer SiHa cells (0.6 × 106) were grown in 96-well plates. After treatments, the cells were sloughed off, 25 μL of cell culture was put onto glass slides and subjected to staining with 1 μL of AO and PI. The slides were examined with a fluorescent microscope. For cell cycle analysis, the SiHa cells were incubated with varying concentrations of Res medium and/or cis-DDP (1, 5, 10, 15, 20 μmol/L or μg/ml) for 24 h. The cells were then washed with phosphate-buffered saline (PBS) and stained with propidium iodide (PI). The distribution of the cells in the cell cycle phases was assessed by using the FACS flow cytometer.

### Reactive Oxygen Species (ROS) and Mitochondrial Membrane Potential (ΔΨm) Detection

Intracellular ROS were detected by using the H2DCF-DA fluorescent probe with a fluorescence microscope; the laser wavelength range was 460–490 nm. The fluorescence reading was processed and analyzed; the ROS expression was calculated as fluorescence density*10^3^/mg protein. ΔΨm was detected with the Mitochondrial Membrane Potential Assay Kit (JC-1) (Genmed, Shanghai, China). Briefly, 100 μL of JC-1 dye was added to the frozen slide at 37°C for 20 min in darkness. The excess dye was washed away with JC-1 staining buffer and then rinsed three times with PBS. Observations were made immediately with a confocal microscope. For each slide, four different fields were randomly selected, and the average intensity of red and green fluorescence was recorded (in live cells, the mitochondria appear red with absorption/emission maxima of 585/590 nm, and in apoptotic and dead cells, they were green with absorption/emission maxima of 510/530 nm). A detailed protocol is available (Zhou et al., 2014).

### Spectrophotography

The enzyme activities and H_2_O_2_ content were detected with commercially available kits (JianCheng Bioengineering Institute, Nanjing, China). Spectrophotography was used to calculate the enzyme activity (MnSOD, SOD-1, CAT, and GPx) and the H_2_O_2_ content. The detailed protocol is available elsewhere (Kumar et al., 2020).

### Western Blotting

The SiHa cells were first washed with cold PBS and suspended in lysis buffer at 4 °C and then transferred to 95 °C. The protein content of each cell extract was then checked by the Bradford assay. For each sample, 40 μg of protein were loaded and separated by SDS-PAGE before being shifted to the polyvinylidene fluoride membrane. The membranes were treated with Tris-buffered saline (TBS) and exposed to primary antibodies at 4 °C. Then, the membranes were washed with TBST buffer three times and were incubated with the corresponding horseradish peroxidase-conjugated secondary antibody and goat anti-rabbit IgG (1:500 dilution) for 1 h at room temperature and developed using the ECL substrate. Mouse monoclonal anti-GAPDH (1:3,000 dilution) was used as loading controls.

### Statistics

Data were shown as mean ± standard deviation. Statistical analysis was performed using Student’s t-test with SPSS 19.0 software. *p* values < 0.05 were taken as indicative of a significant difference.

## Results

### Combination of RES and Cis-DDP Enhanced Apoptosis in SiHa Cells and Inhibited Cell Proliferation and Caused S Cell Cycle Arresting

There were four different group settings in this study: vehicle control, cis-DDP, RES, and RES + cis-DDP. Each group was tested with various concentrations, and optimum conditions were chosen. The MTT assays were performed to calculate the CDI of each group, as shown in Supplementary Table S1; the CDIs of RES + cis-DDP combinations were all less than one in all groups, and it suggested a synergistic effect between RES and cis-DDP. The optimum concentration for this study was chosen as 5uM of RES and 5ug/ml of cis-DDP treatment for 48 h when the CDI is the lowest among all groups. The cell morphological changes were observed with an inverted microscope during the study (Supplementary Figure S1), and the RES + cis-DDP group showed more irregular-shaped cells with poor refraction and more apoptotic bodies compared with other groups, suggesting that the RES + cis-DDP combination compromised the morphology of the SiHa cells.

**FIGURE 1:**
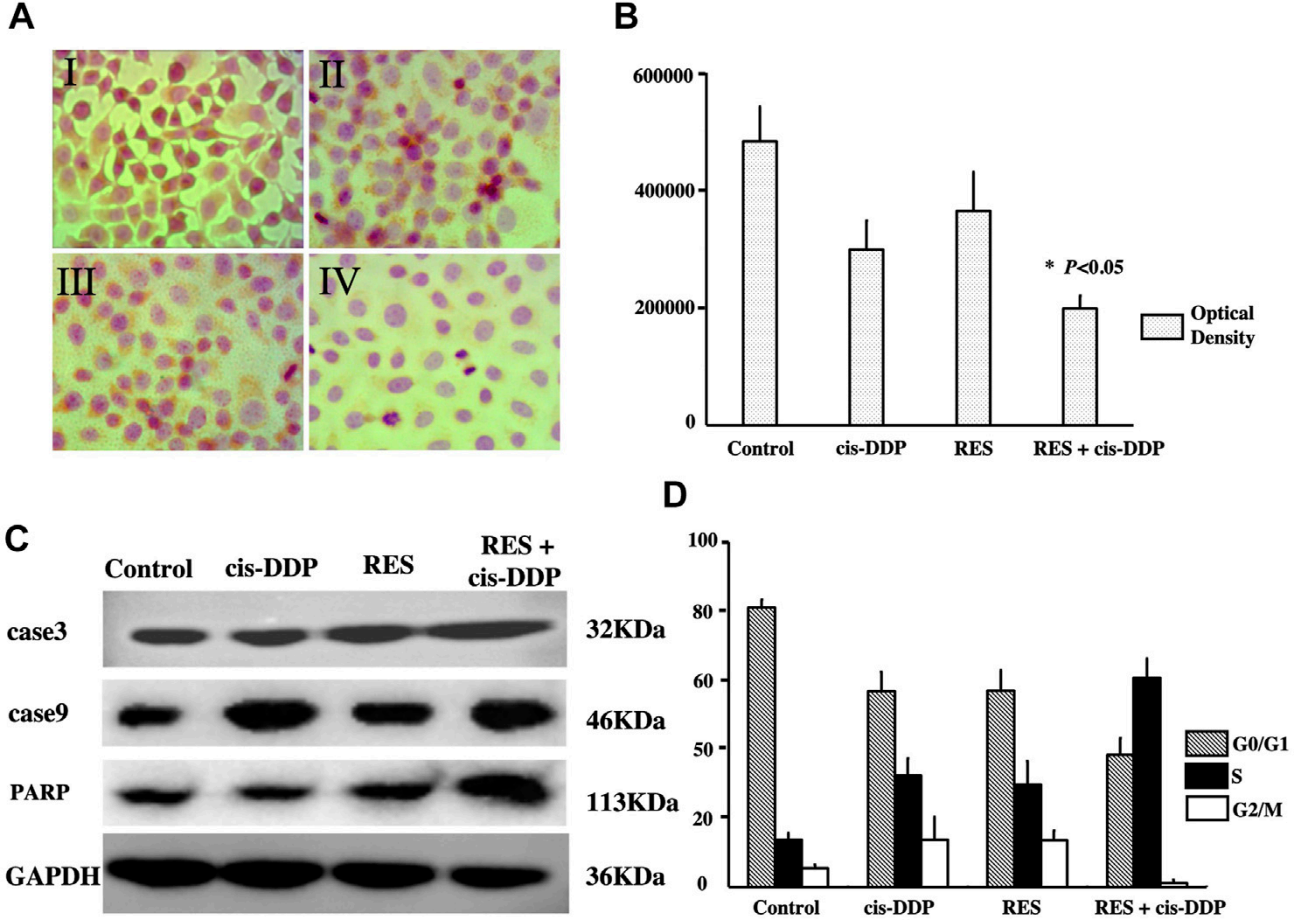
RES + cis-DDP enhanced apoptosis in SiHa cells by inhibition of cell proliferation and S cell cycle arresting. **(A)** Images of proliferating cell nuclear antigens (PCNAs) in SiHa cells by the IHC method. I, II, III, and IV represent vehicle control, cis-DDP, RES, and RES + cis-DDP, respectively. PCNAs were dark red spots in the nucleus. **(B)** Expression of PCNA by the IHC method was recorded as optical density, and it showed RES + cis-DDP significantly reduced the PCNA expression compared with other groups (*p < 0.05). **(C)** Expression of PARP and cleaved caspases 3 and 9 in each group was evaluated by Western blotting, and RES + cis-DDP caused a considerable increase in the expression of PARP and cleaved caspases 3 and 9 among all groups. **(D)** Impact of the treatments on the distribution of SiHa cells in cell cycle phases was assessed by FACS. RES + cis-DDP increased the percentage of the SiHa cells in the S phase of the cell cycle.

To investigate the mechanism of the morphological changes, the proliferating cell nuclear antigens (PCNA) were detected, and cell apoptosis was evaluated. As is shown in Figures 1A, B, the expression of proliferating cell nuclear antigens (PCNA) was detected in all groups. The PCNA expression was significantly lower in the RES + cis-DDP group than that in other single treatment groups (*p* < 0.05), indicating that RES + cis-DDP inhibited cell proliferation considerably. FACS apoptosis assays were performed to detect the apoptosis rate in different groups, and the RES + cis-DDP group showed the highest apoptosis rate among all groups (Supplementary Table S2). The treatments also altered the expression of the PARP and cleaved caspases 3 and 9 expressions in SiHa cancer cells, and RES + cis-DDP caused a considerable increase in the expression of PARP and cleaved caspases 3 and 9 among all groups (Figure1Cfig1).

The impact of RES treatments on the distribution of SiHa cells in various cell cycle phases was assessed by FACS. It was found that RES + cis-DDP caused a remarkable increase in the percentage of the SiHa cells in the S phase of the cell cycle. The percentage of SiHa cells in the S phase increased from 38.01% in the cis-DDP-alone group to 66.37% upon treatment with RES + cis-DDP (Figure 1D; Supplementary Table S3; Supplementary Figure S2). These results indicated that RES + cis-DDP induced S cell cycle arrest of the SiHa cervical cancer cells.

### RES + Cis-DDP Combination Decreased the ROS Expression and Enhanced Mitochondrial Transmembrane Potential (ΔΨm) in SiHa Cells

**FIGURE 2:**
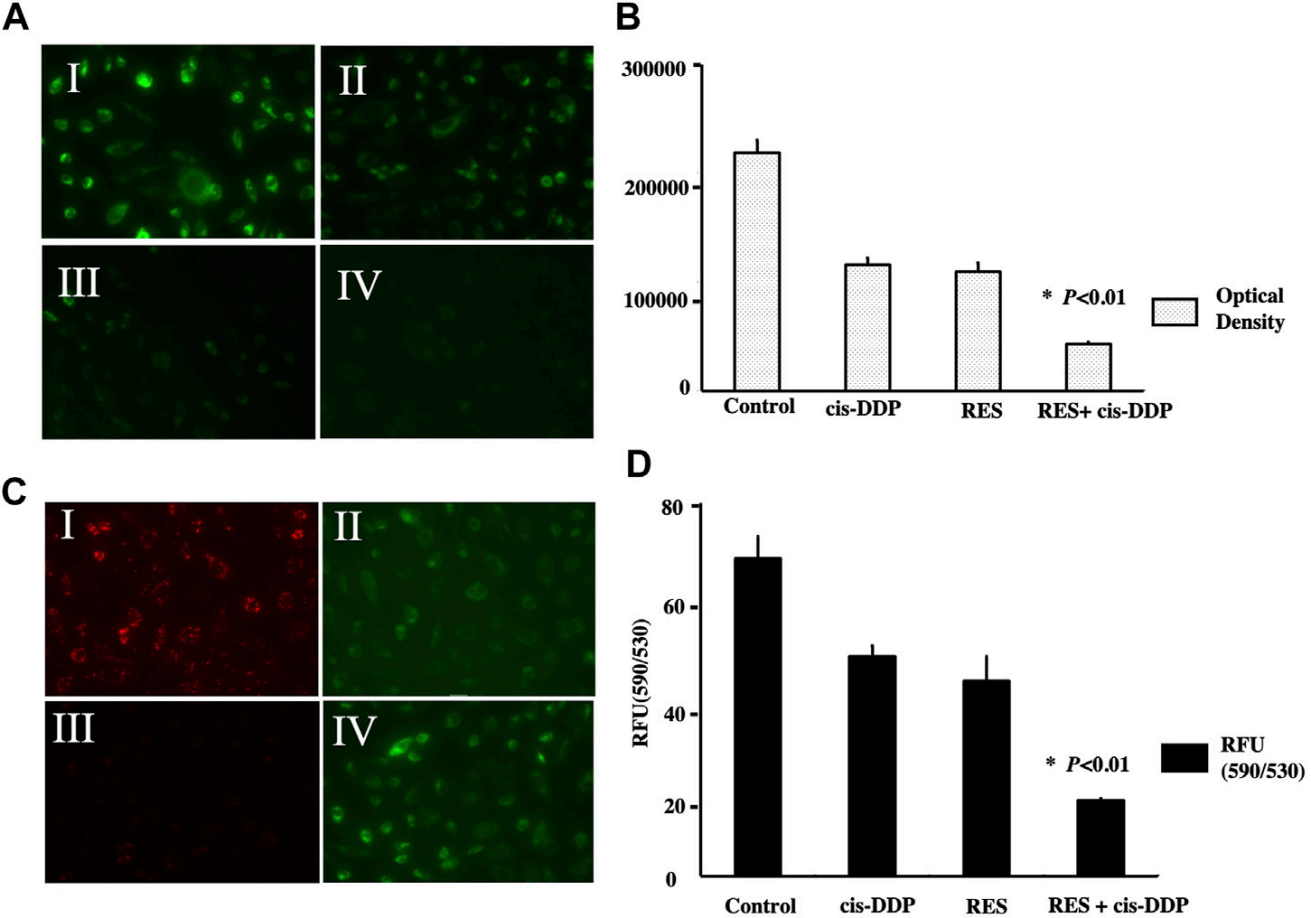
Res + cis-DPP decreased the ROS content and enhanced mitochondrial transmembrane potential (ΔΨm) in SiHa cells. **(A)** Intracellular ROS were detected by H2DCF-DA fluorescent probe (green) with a fluorescence microscope, and the laser output wavelength range was 460–490 nm with 100 mW/cm^2^ power density. Images I, II, III, and IV represent vehicle control, cis-DDP, RES, and RES + cis-DDP, respectively. **(B)** Intracellular DCF fluorescence (an indicator of ROS formation) was recorded as the optical density, and it showed that RES + cis-DDP had the lowest ROS expression among all groups (*p < 0.01). **(C)** Images of the mitochondrial membrane potential assay (JC-1 method). Images I, II, III, and IV represent vehicle control, cis-DDP, RES, and RES + cis-DDP, respectively. In cells with high ΔΨm; the JC-1 was accumulated and appeared red color under a fluorescence microscope (image c-I). In cells with low ΔΨm, the less concentrated JC-1 showed green color (images c II and IV). **(D)** JC-1 fluorescence was recorded and converted into relative fluorescence units (RFU), and it showed that RES and/or cis-DDP significantly reduced ΔΨm compared with the control group; the RES + cis-DDP group had the lowest ΔΨm among all groups (*p* < 0.01)

Intracellular ROS were detected by the H2DCF-DA fluorescent probe with a fluorescence microscope at wavelength 460–490 nm. The optical density in each group was calculated by spectrophotography to quantify the ROS expression, and it showed that RES + cis-DDP had the lowest ROS expression among all groups (*p* < 0.01, Figures 2A, B)

As is shown in Figure 2C, the SiHa cells in the control group were mostly live cells and appeared red (Figure 2 c-I); in cis-DDP and RES + cis-DDP groups, the majority of cells were apoptotic or dead, so the color was green (Figure 2 c-II, IV). ΔΨm of SiHa cells in different groups was detected with mitochondrial membrane potential assay. It showed that RES and/or cis-DDP significantly reduced ΔΨm compared with the control group, and the RES + cis-DDP group had the lowest ΔΨm among all groups (*p* < 0.01, Figure 2D)

### RES + Cis-DDP Combination Enhanced the Expression of SIRT3, and Silencing of the *SIRT3* Gene Compromised RES + cis-DDP–Induced SiHa Inhibition

**FIGURE 3:**
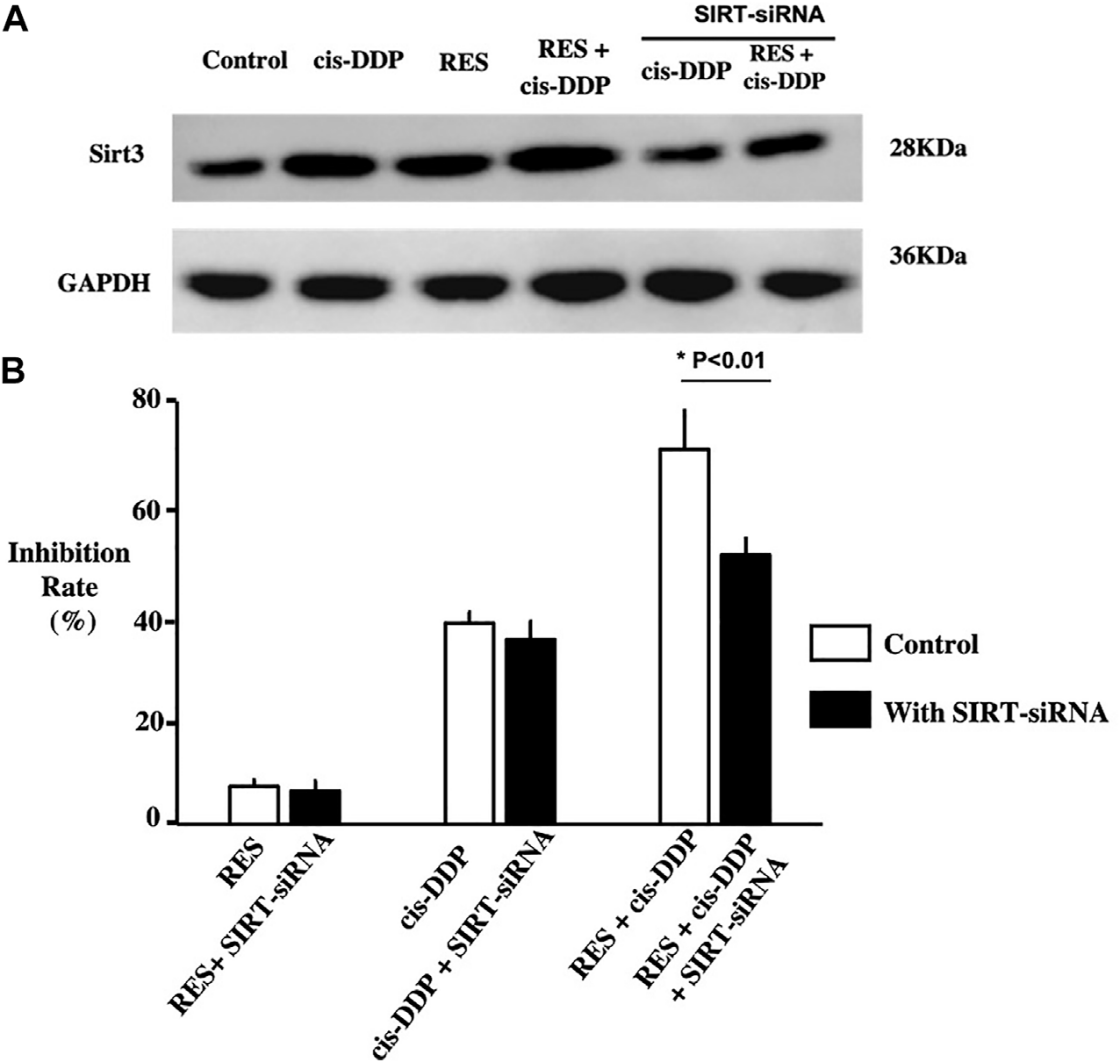
Res combined with cis-DPP enhanced the expression of SIRT3; silencing of SIRT3 compromised Res + cis-DPP–induced SiHa inhibition. **(A)** Western blotting of the SIRT3 expression in each group showed that RES + cis-DPP significantly enhanced the SIRT3 expression compared with other groups (*p* < 0.05). **(B)** Normalized growth rate inhibition of the RES alone, cis-DDP alone, and RES + cis-DDP groups, with or without silencing of SIRT3. It showed that SIRT3 RNA silencing significantly compromised the SiHa growth inhibition rate only in the RES + cis-DDP group (*p* < 0.01).

To study whether the SIRT-3 relative anti-oxidative pathway plays a role in the anti-cancer effect of RES and cis-DDP combination, SIRT-3 expression in each group was tested with Western blotting. The RES + cis-DDP group had the highest SIRT-3 expression among all groups (Figure 3A). SIRT3 RNA silencing significantly compromised the SiHa growth inhibition rate only in the RES + cis-DDP group (*p* < 0.01, Figure 3B; Supplementary Table S4).

### RES + cis-DDP Combination Enhanced the Expression and Activation of Antioxidant Enzymes and Increased the Intracellular H_2_O_2_, and Silencing of *SIRT3* Gene Decreased the MnSOD Expression and Intracellular H_2_O_2_ in the RES + Cis-DDP Group

**FIGURE 4:**
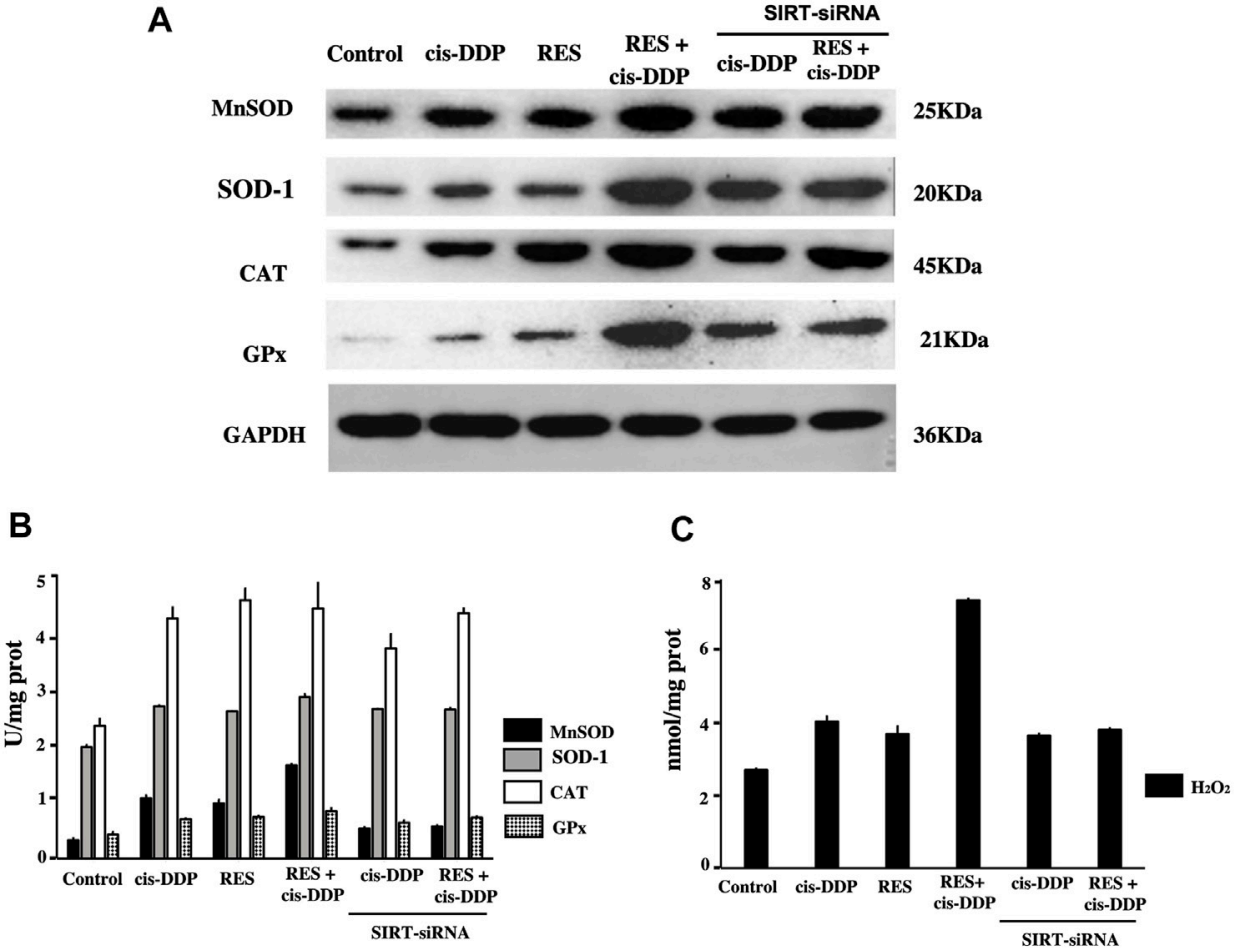
RES + cis-DDP combination enhanced the expression and activation of antioxidant enzymes and increased the intracellular H_2_O_2_; silencing of the SIRT3 gene decreased the MnSOD expression and intracellular H_2_O_2_ in the RES + cis-DDP group. **(A)** Expression of antioxidant enzymes (MnSOD, SOD-1, CAT, and GPx) in each group was evaluated by Western blotting. RES + cis-DDP enhanced the expressions of MnSOD, SOD-1, CAT, and GPx compared with other groups (*p* < 0.05), and SIRT3 interference significantly decreased the MnSOD expressions in the RES + cis-DDP group (*p* < 0.01); SOD-1, CAT, and GPx was less impacted by SIRT3 silence. **(B)** Antioxidant enzyme activities were evaluated in each group, and silencing of SIRT3 was performed in the cis-DDP alone group and the RES + cis-DDP group. **(C)** H_2_O_2_ content in each group was evaluated, and silencing of SIRT3 was performed in the cis-DDP alone group and the RES + cis-DDP group.

The expression of antioxidant enzymes (MnSOD, SOD-1, CAT, and GPx) in each group was tested by Western blotting (Figure 4A). RES + cis-DDP significantly enhanced the expression of these enzymes compared with RES or cis-DDP alone (*p* < 0.05). SIRT3 interference significantly decreased the MnSOD expression in the RES + cis-DDP group (*p* < 0.01). The enzyme activities were detected with commercially available kits. As shown in Figure 4B, compared with control, all treatments (cis-DDP alone, RES alone, and RES + cis-DDP) enhanced the activities of antioxidant enzymes (MnSOD, SOD-1, CAT, and GPx). Enzyme activities of MnSOD in the cis-DDP alone group and the RES + cis-DDP group significantly decreased after SIRT3 interference (*p* < 0.05, Figure 4B). RES + cis-DDP greatly enhanced the intracellular H_2_O_2_, and the enhancement of H_2_O_2_ can be blocked by SIRT3 interference (*p* < 0.01, Figure 4C). No obvious change of H_2_O_2_ was found in the cis-DDP alone and RES alone groups before or after SIRT3 interference.

## Discussion

Cervical cancer is one of the most common types of cancer in women, and it ranks second in the incidence of all cancers globally (Motoki et al., 2015; Schiffman, 2017). The frequency of cervical cancer differs across the world, with more than 85% of deaths occurring in underdeveloped countries (Zhang et al., 2021). The cis-DDP-based chemotherapies were the first-line treatment for late-stage cervical cancer (Gadducci and Cosio, 2020). Considering the limited clinical outcomes in advanced-stage patients and the side effects of chemotherapies (Sharma et al., 2020), the identification of novel and safer anti-cancer molecules that may act as synergistic agents is required in the treatment of cervical cancer.

RES is a natural polyphenol, and it has been confirmed to have a broad range of biological activities, including anti-cancer effects in many tumors (Jiang et al., 2017; Mukherjee et al., 2017). Recently, RES was found to be able to suppress the transcription and expression of HPV E6 and E7 genes in HeLa cells and inhibit the progression of cervical cancer (Kim et al., 2012; Sun et al., 2021). RES was reported to interrupt the G1/S phase transition in MCF-7, HeLa, and ca Ski cells and induce the G1/S arrest in human prostate cancer cell lines (Wang et al., 2010; Medina-Aguilar et al., 2016; Sun et al., 2021). In the present study, it was found that RES inhibited the growth of the SiHa cell lines, and when RES was combined with cis-DDP (RES + cis-DDP), it could significantly enhance cell apoptosis and increase the proportion of G1 phase cells and decrease the proportion of the S phase in cells. These data suggest that RES promotes the G1/S arrest in SiHa cells. The induction of G1/S arrest can be a possible mechanism through which RES inhibits the development of cervical cancer. These results were consistent with those of previous studies (Sun et al., 2021). To the best of our knowledge, this is the first study to test the anti-cancer effect of RES + cis-DDP in SiHa cervical cell lines.

SIRT-3 is a protein deacetylase localized in the mitochondria and regulates mitochondrial function (Ansari et al., 2017). Studies had shown that SIRT3 can be directly activated by RES, and the activated SIRT3 may play a role in the RES biological functionality (McDonnell et al., 2015; Bagul et al., 2018). In our study, SIRT3 silencing significantly compromised the growth inhibition in the RES + cis-DDP group, and it suggested that the anti-cancer effect of RES + cis-DDP was SIRT3-dependent. It was known that SIRT-3 regulated the key enzyme activities for acetylation and reactive oxygen species (ROS) detoxification (Ilari et al., 2020). Since ROS is one of the important factors to impact the occurrence and development of tumors (Tudek et al., 2010; McDonnell et al., 2015), we presumed that RES + cis-DDP inhibits the growth of SiHa cells by regulating the ROS expression through the SIRT3-dependent pathway. Indeed, it was found in our study that the RES + cis-DDP combination significantly enhanced the expression of SIRT3 and decreased the ROS content compared with other groups.

MnSOD is an antioxidant enzyme that catalyzed the dismutation of superoxide to H_2_O_2_ in the mitochondria, and the expression of MnSOD is particularly important in the cellular redox reaction. In our study, the RES + cis-DDP combination significantly enhanced the expression and enzyme activity of MnSOD and increased the H_2_O_2_ content, while other antioxidant enzymes such as CAT and GPx did not show such statistical significance. By SIRT3 silencing, the MnSOD expression and enzyme activity along with H_2_O_2_ content were all significantly reduced in the RES + cis-DDP group. MnSOD was found to be able to convert superoxide radicals into H_2_O_2_ and molecular oxygen (Candas and Li, 2014; Fan et al., 2019); the elevated MnSOD activity and expression could be a reason for decreased ROS and elevated H_2_O_2_ in the RES + cis-DDP groups. Our data suggested that RES may directly activate SIRT3 to regulate MnSOD, increase the content of H_2_O_2_, promote cell apoptosis, and increase the efficacy of cis-DDP.

## Conclusion

Our data showed that the synergistic effect of RES and cis-DDP was SIRT3-dependent and related to MnSOD activation and increased the H_2_O_2_ content. Our study suggested that RES may act as a potential synergistic agent to enhance the anti-cancer effect of cis-DDP in the treatment of cervical cancer.

## Data Availability

The original contributions presented in the study are included in the article/Supplementary Material; further inquiries can be directed to the corresponding author.
