# Supplementary figures and images for: Resveratrol Enhances the Anti-Cancer Effects of Cis-Platinum on Human Cervical Cancer Cell Lines by Activating the SIRT3 Relative Anti-Oxidative Pathway
Source: Front Pharmacol. 2022 Jul 5;13:916876. doi: 10.3389/fphar.2022.916876 (PMC9294406; doi:10.3389/fphar.2022.916876)

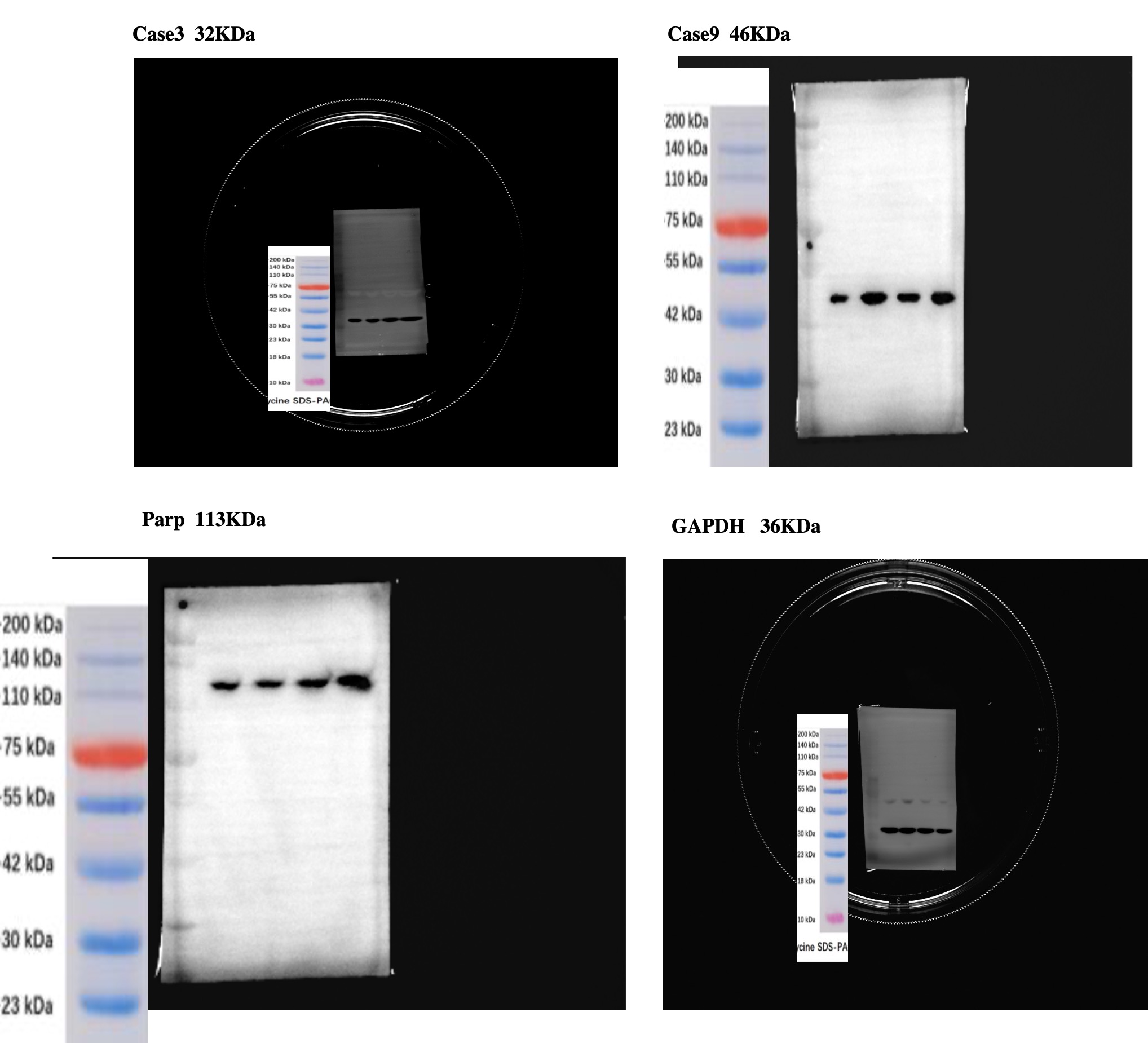

Supplement: Supplementary file 1 [file Image3.JPEG]

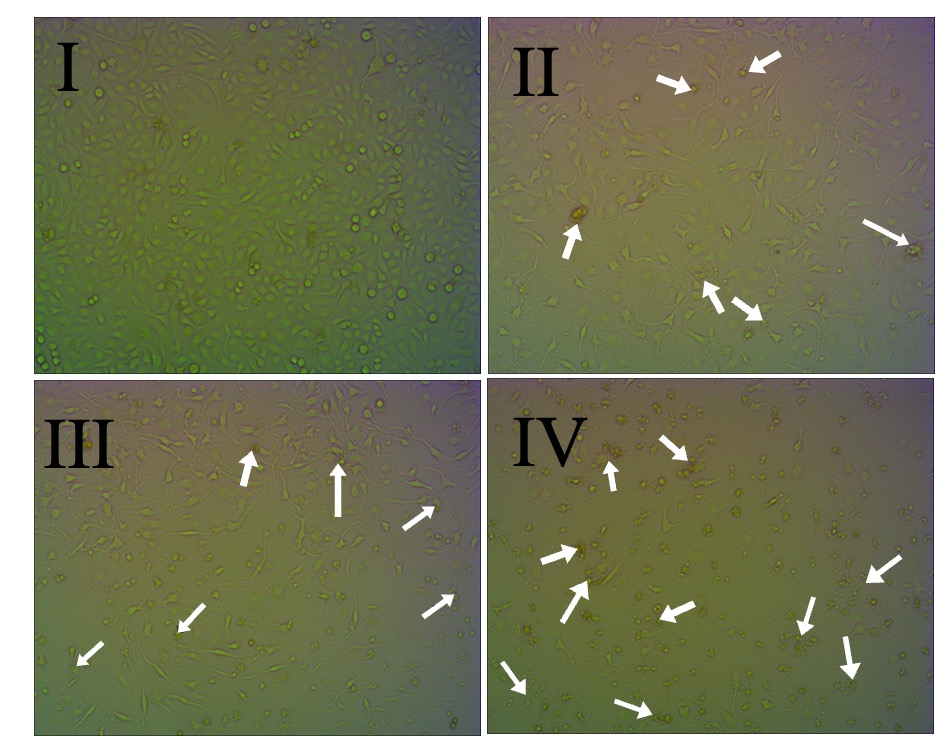

Supplement: Supplementary file 3 [file Image1.JPEG]

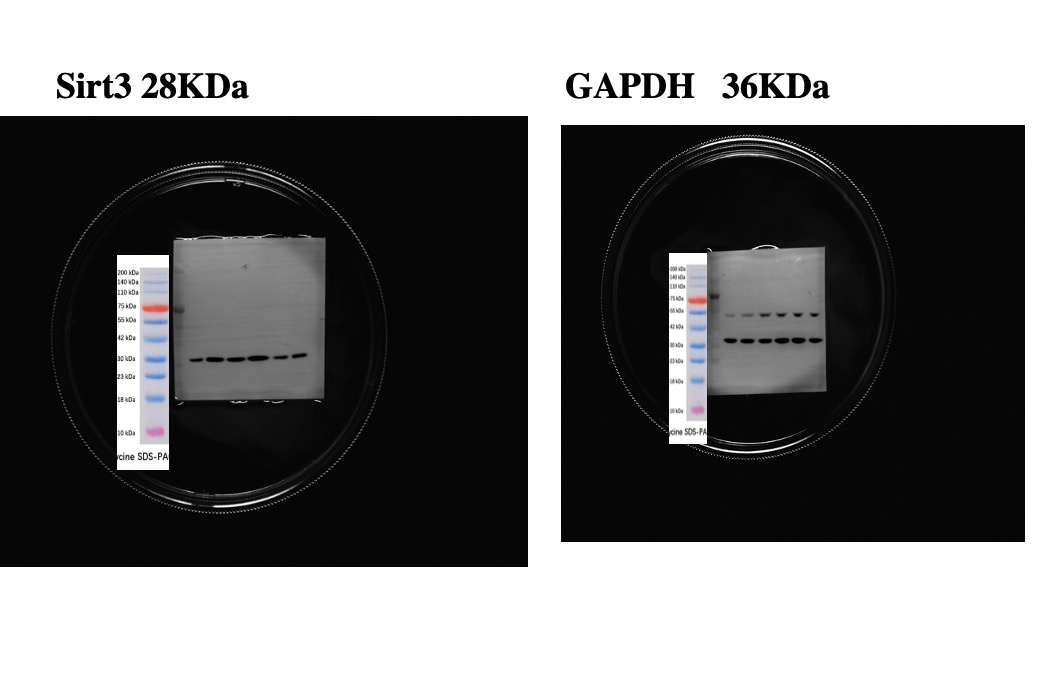

Supplement: Supplementary file 4 [file Image4.JPEG]

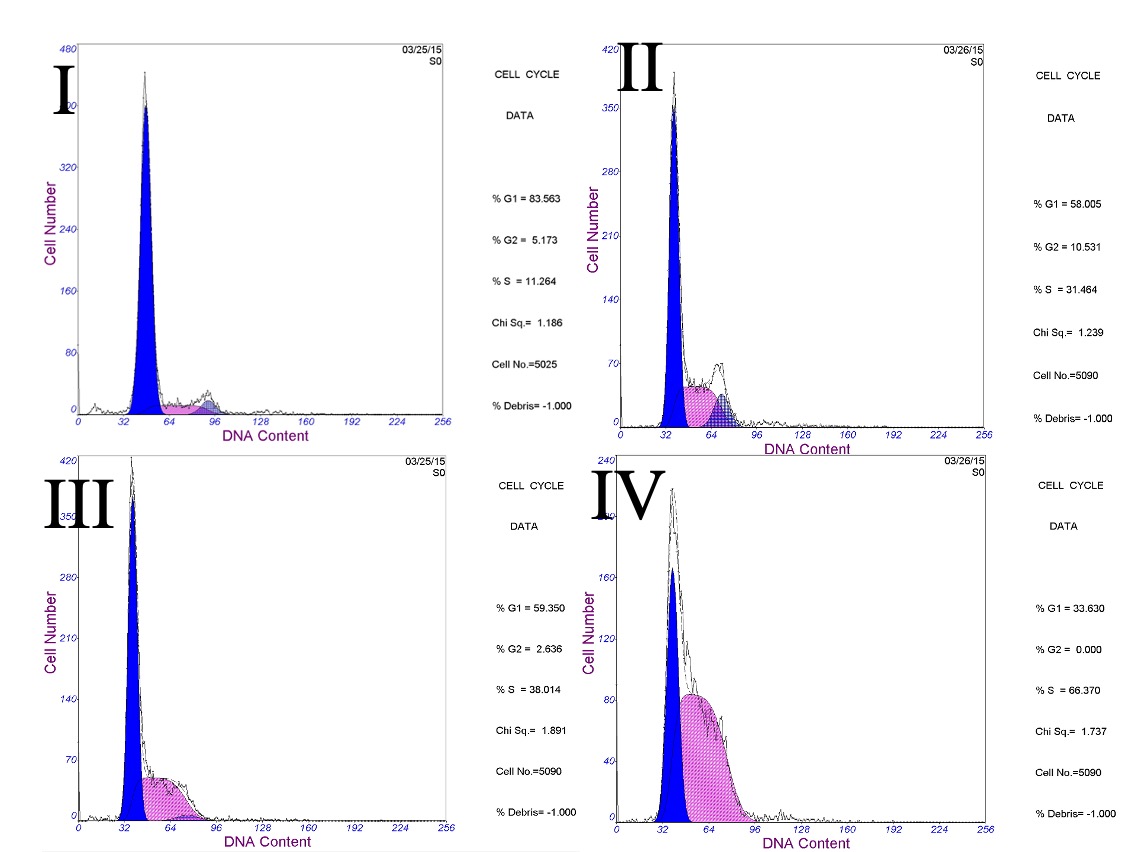

Supplement: Supplementary file 5 [file Image2.JPEG]

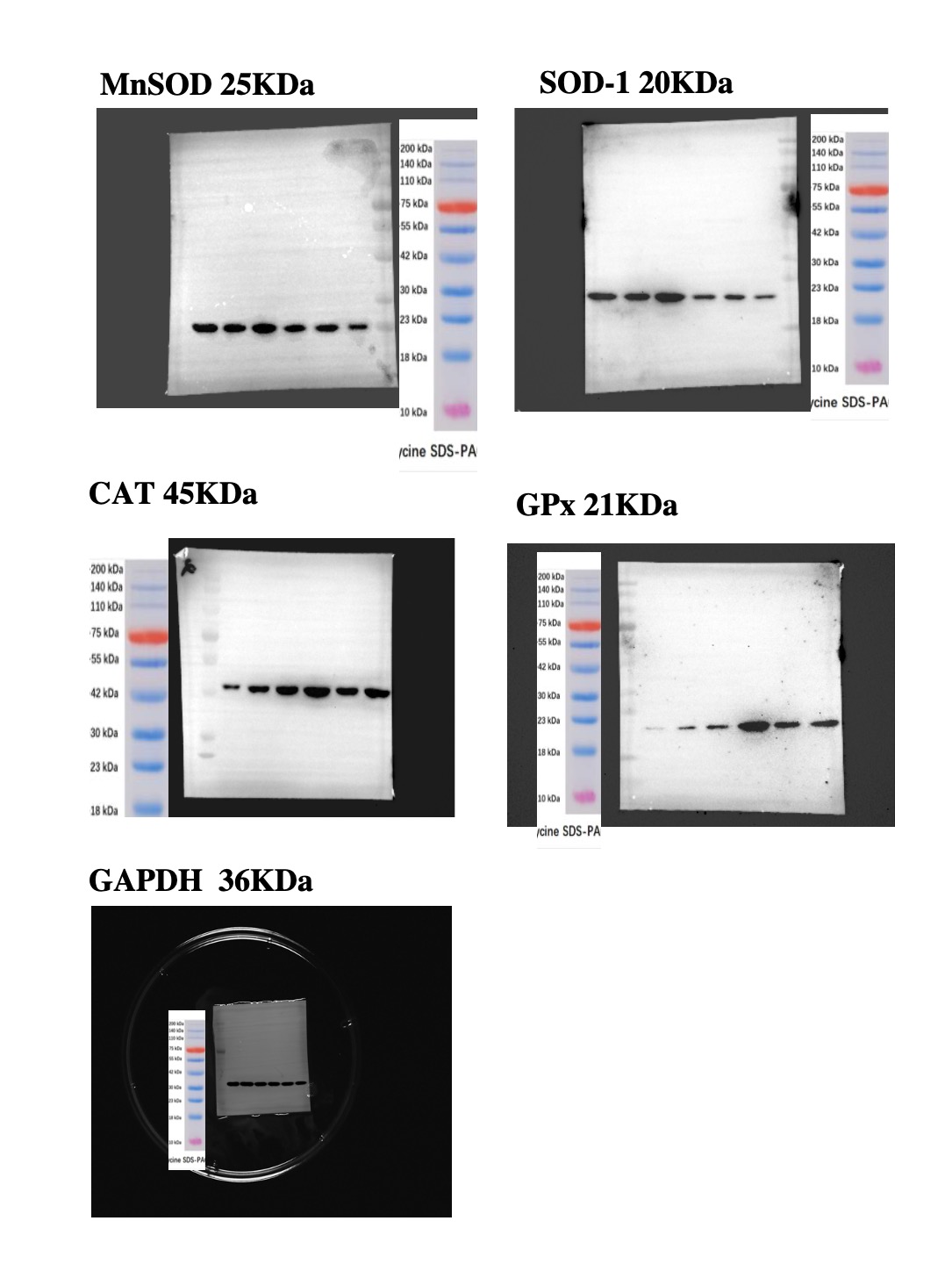

Supplement: Supplementary file 6 [file Image5.JPEG]
