# Supplementary material for: Resveratrol Enhances the Anti-Cancer Effects of Cis-Platinum on Human Cervical Cancer Cell Lines by Activating the SIRT3 Relative Anti-Oxidative Pathway
Source: Front Pharmacol. 2022 Jul 5;13:916876. doi: 10.3389/fphar.2022.916876 (PMC9294406; doi:10.3389/fphar.2022.916876)
Supplement: Supplementary file 2 [file Table1.DOCX]

**Supplementary Table 1** Effects on the growth of SiHa cells with different concentrations of RES and cis-DDP combinations by MTT assay in 24 and 48 hours

| Concentration  of each reagent | 24 hours | | | | 48 hours | | | |
| --- | --- | --- | --- | --- | --- | --- | --- | --- |
| Cis-DDP RES | Mean ± SD | Inhibition rate （%） | | CDI value | Mean ± SD | Inhibition rate （%） | | CDI value |
| 1μg/ml 1μM  1μg/ml 5μM  1μg/ml 10μM  5μg/ml 1μM  5μg/ml 5μM  5μg/ml 10μM  10μg/ml 1μM  10μg/ml 5μM  10μg/ml 10μM | 0.322±0.027  0.294±0.023  0.265±0.059  0.288±0.043  0.270±0.027  0.243±0.016  0.247±0.027  0.223±0.058  0.206±0.025 | 15.95  25.08  34.65  27.06  33.00  42.02  40.07  48.40  54.02 | 0.960  0.902  0.853  0.956  0.922  0.869  0.904  0.842  0.816 | | 0.363±0.026  0.299±0.032  0.249±0.041  0.199±0.034  0.157±0.052  0.173±0.021  0.092±0.030  0.100±0.035  0.078±0.009 | | 8.17  27.55  42.87  58.09  70.88  65.91  90.56  88.03  94.93 | 1.050  0.906  0.864  0.763  0.630  0.798  0.843  0.962  0.854 |

* All CDI values were less than 1, and in the group of RES5μM and cis-DD 5μg/ml, the CDI value in 48 hours was 0.630 and it was the lowest CDI among all groups

**Supplementary Table 2**

The apoptosis rate of SiHa cells detected by flow cytometry in each group (%)

| Groups | LR(%) | UR(%) | Apoptosis rate(%) |
| --- | --- | --- | --- |
| Control  RES  cis-DDP  RES+cis-DDP | 2.65  5.12  6.90  21.63 | 0.36  11.64  13.7  31.74 | 3.01  16.76  20.6  53.37 |

* UL(necrotic cells), LL(survival cells), LR(early apoptotic cells), UR(later apoptotic cells); all apoptotic cells is LR+UR.

**Supplementary Table 3**

The proportion of cell cycle phases detected by flow cytometry (%)

| Groups | G0/G1 | S | G2/M | PI（%） |
| --- | --- | --- | --- | --- |
| Control  RES  cis-DDP  RES+cis-DDP | 83.563  59.35  58.005  33.630 | 11.264  38.014  31.464  66.370 | 5.173  10.531  10.531  0 | 16.437  40.650  41.995  66.370 |

**Supplementary Table 4**

Detection of inhibitory rate before and after the silence of SIRT3 by MTT method

| Groups | 24h | | 48h | |
| --- | --- | --- | --- | --- |
|  | Mean ± SD | inhibitory rate (%) | Mean ± SD | inhibitory rate (%) |
| RES  RES/siRNA  cis-DDP  cis-DDP/siRNA  RES+cis-DDP  RES+cis-DDP/  SiRNA | 0.353±0.017  0.370±0.020  0.307±0.020  0.325±0.037  0.270±0.027  0.289±0.036 | 5.72  5.71  20.68  18.30  33  29.73 | 0.366±0.014  0.477±0.022  0.265±0.037  0.352±0.024  0.157±0.052  0.283±0.010 | 7.15  6.25  38.00  34.93  70.88  50.87 |

**Supplementary Figure Legends**

**Supplementary figure 1**

The images of cell morphology under inverted microscope in each group. I, II, III, IV represents the group of vehicle control, cis-DDP, RES, and RES+ cis-DDP, respectively. and RES + cis-DDP group (IV) showed more irregular shaped cells with poor refraction and more apoptotic bodies (pointed out by white arrows) compared with other groups.

**Supplementary figure2**

Effect of treatment on the distribution of the cell cycle arrest of the SiHa cells as depicted by flow cytometry. I, II, III, IV represents the group of vehicle control, cis-DDP, RES, and RES+ cis-DDP, respectively. The figure shows that RES+ cis-DDP induces S arrest of the SiHa cells. The experiments were performed in triplicate and representative images were chosen.
